# GLP-1 receptor agonist-based therapies and cardiovascular risk: a review of mechanisms

**DOI:** 10.1530/JOE-24-0046

**Published:** 2024-09-19

**Authors:** Neerav Mullur, Arianne Morissette, Nadya M Morrow, Erin E Mulvihill

**Affiliations:** 1 The University of Ottawa, Faculty of Medicine, Ottawa, Ontario, Canada; 2 The University of Ottawa Heart Institute, Ottawa, Ontario, Canada; 3 Department of Biochemistry, Microbiology and Immunology, The University of Ottawa, Faculty of Medicine, Ottawa, Ontario, Canada

**Keywords:** cardiovascular, hormone action, incretins, metabolism, nutrition

## Abstract

Cardiovascular outcome trials (CVOTs) in people living with type 2 diabetes mellitus and obesity have confirmed the cardiovascular benefits of glucagon-like peptide 1 receptor agonists (GLP-1RAs), including reduced cardiovascular mortality, lower rates of myocardial infarction, and lower rates of stroke. The cardiovascular benefits observed following GLP-1RA treatment could be secondary to improvements in glycemia, blood pressure, postprandial lipidemia, and inflammation. Yet, the GLP-1R is also expressed in the heart and vasculature, suggesting that GLP-1R agonism may impact the cardiovascular system. The emergence of GLP-1RAs combined with glucose-dependent insulinotropic polypeptide and glucagon receptor agonists has shown promising results as new weight loss medications. Dual-agonist and tri-agonist therapies have demonstrated superior outcomes in weight loss, lowered blood sugar and lipid levels, restoration of tissue function, and enhancement of overall substrate metabolism compared to using GLP-1R agonists alone. However, the precise mechanisms underlying their cardiovascular benefits remain to be fully elucidated. This review aims to summarize the findings from CVOTs of GLP-1RAs, explore the latest data on dual and tri-agonist therapies, and delve into potential mechanisms contributing to their cardioprotective effects. It also addresses current gaps in understanding and areas for further research.

## Introduction

The incretin concept stems from the observation that insulin levels are higher following the intake of glucose through the gastrointestinal tract compared to an isoglycemic exposure to the islet through intravenous glucose administration (McIntyre *et al.* 1965). The incretin hormones, glucose-dependent insulinotropic polypeptide (GIP), and glucagon-like peptide-1 (GLP-1) potentiate meal-stimulated insulin secretion through direct and indirect actions on islet β-cells. The first incretin hormone to be identified, GIP, is mainly synthesized within the enteroendocrine K cells of the duodenum and jejunum. Nutrients in the gut lumen, including glucose, fatty acids, and amino acids, stimulate GIP secretion, rapidly increasing its circulating levels (Brown & Dryburgh 1971, Jorsal *et al.* 2018). The majority of circulating GLP-1 is produced and secreted by the enteroendocrine L cells located in the small intestine and colon, with the highest abundance of GLP-1-producing cells in the ileum in rodents and the colon in humans (Eissele *et al.* 1992, Song *et al.* 2019). GIP and GLP-1 are also synthesized in the brain. GIP is detected at high levels in the rat hippocampal dentate gyrus, olfactory bulb, and cerebellum, and at lower levels in the brainstem, cerebral cortex, amygdala, substantia nigra, thalamus, and lateral septal nucleus, frequently colocalizing with neuronal markers (Nyberg *et al.* 2005, 2007). On the other hand, GLP-1 is likely produced by proglucagon-expressing neurons predominantly in the caudal nucleus tractus solitarius of the brainstem (Jin *et al.* 1988, Llewellyn-Smith *et al.* 2011). It is difficult to determine with certainty which products of proglucagon are secreted, due to the chemogenetic methods used to target these neurons (Drucker 2018). Moreover, increasing evidence postulates a production and paracrine role for small amounts of bioactive GLP-1 produced by pancreatic α-cells (Nie *et al.* 2000, Kilimnik *et al.* 2010, de Souza *et al.* 2020).

Both incretin hormones exert a glucose-lowering action through two distinct yet structurally related class B G-protein-coupled receptors expressed in most pancreatic β cells. *Gipr* mRNA is also detected in both α cells and δ cells of the pancreas (DiGruccio *et al.* 2016). Low levels of GLP-1 receptor (GLP-1R) have also been detected in pancreatic δ cells, whereas the detection of expression in α-cells has been inconsistent (DiGruccio *et al.* 2016, Ast *et al.* 2020). Beyond the pancreas, the GIP receptor (GIPR) is expressed within the pericytes and mesothelial cells of the adipose tissue (Campbell *et al.* 2022), heart myocytes and pericytes (Ussher *et al.* 2018), vascular endothelial cells (Usdin *et al.* 1993, Zhong *et al.* 2000), and central nervous system (neurons, glia, pericytes, and oligodendrocytes) (Adriaenssens *et al.* 2019, Ludwig *et al.* 2021), as well as bone osteocytes, osteoblasts, osteoclasts, myeloid cells, and T cells (Bollag *et al.* 2000, Zhong *et al.* 2007, Pujadas *et al.* 2020). GLP-1R is expressed in endothelial cells (Kim *et al.* 2013, Pyke *et al.* 2014, Richards *et al.* 2014, McLean *et al.* 2022), circulatory system (endothelial cells, vascular smooth muscle cells), kidney vascular smooth muscle cells (Pyke *et al.* 2014, Jensen *et al.* 2015), gut intestinal intraepithelial lymphocytes and enteric neurons (Richards *et al.* 2014, Yusta *et al.* 2015), liver γδ T cells (McLean *et al.* 2021*a*,*b*), and lung endothelial cells (McLean *et al.* 2021*a*,*b*), as well as neurons, astrocytes, and oligodendrocytes in the brain (Merchenthaler *et al.* 1999, Cork *et al.* 2015, Smith *et al.* 2022) and cardiomyocytes (Baggio *et al.* 2018). The actions of GIPR and GLP-1R in the abovementioned cell types have been reviewed previously (Drucker & Holst 2023, Hammoud & Drucker 2023).

## Gut- and pancreas-derived hormone-based therapies for the treatment of cardiometabolic disease

The function of GIP and GLP-1 to reduce glycemia through glucose-dependent potentiation of insulin secretion provided the initial rationale for exploring the feasibility of GLP-1R agonist (GLP-1RA)-based peptide therapies for the management of type 2 diabetes mellitus (T2DM). GLP-1RAs are a class of drugs resistant to degradation by the ubiquitous protease dipeptidyl peptidase-4 (DPP4), known to cleave and inactivate endogenous GLP-1 and GIP. GLP-1RAs have been shown to control glycemia by enhancing insulin secretion in response to hyperglycemia, suppressing glucagon secretion under hyper- or euglycemic conditions, slowing down gastric emptying, and reducing calorie intake and body weight (Nauck *et al.* 2021); the latter three effects have expanded their role beyond diabetes management and are now prescribed for weight loss in individuals with overweight and obesity (Pi-Sunyer *et al.* 2015, Wilding *et al.* 2021). Clinical research over the last decades supports the use of GLP-1RAs in T2DM management (Nauck *et al.* 2021).

People living with T2DM experience a two to four times increase in the incidence of cardiovascular disease (CVD) (Gregg *et al.* 2016), encompassing coronary artery disease, cerebrovascular disease, heart failure (HF), and peripheral artery disease (Carracher *et al.* 2018). Therefore, regulatory guidance specifies that this major cardiovascular risk equivalent requires all new drugs and biologics for glycemic control to demonstrate cardiovascular safety. Cardiovascular outcome trials (CVOTs) in patients living with T2DM have confirmed that GLP-1R agonists decrease cardiovascular mortality and reduce the incidence of myocardial infarction (MI) and stroke. In line with this, the American Diabetes Association and the European Association for the Study of Diabetes updated their guidelines on the management of hyperglycemia and now recommend the use of GLP-1RAs in patients living with T2DM with or without high cardiovascular risk to reduce adverse cardiovascular outcomes (Buse *et al.* 2020).

**Figure 1:**
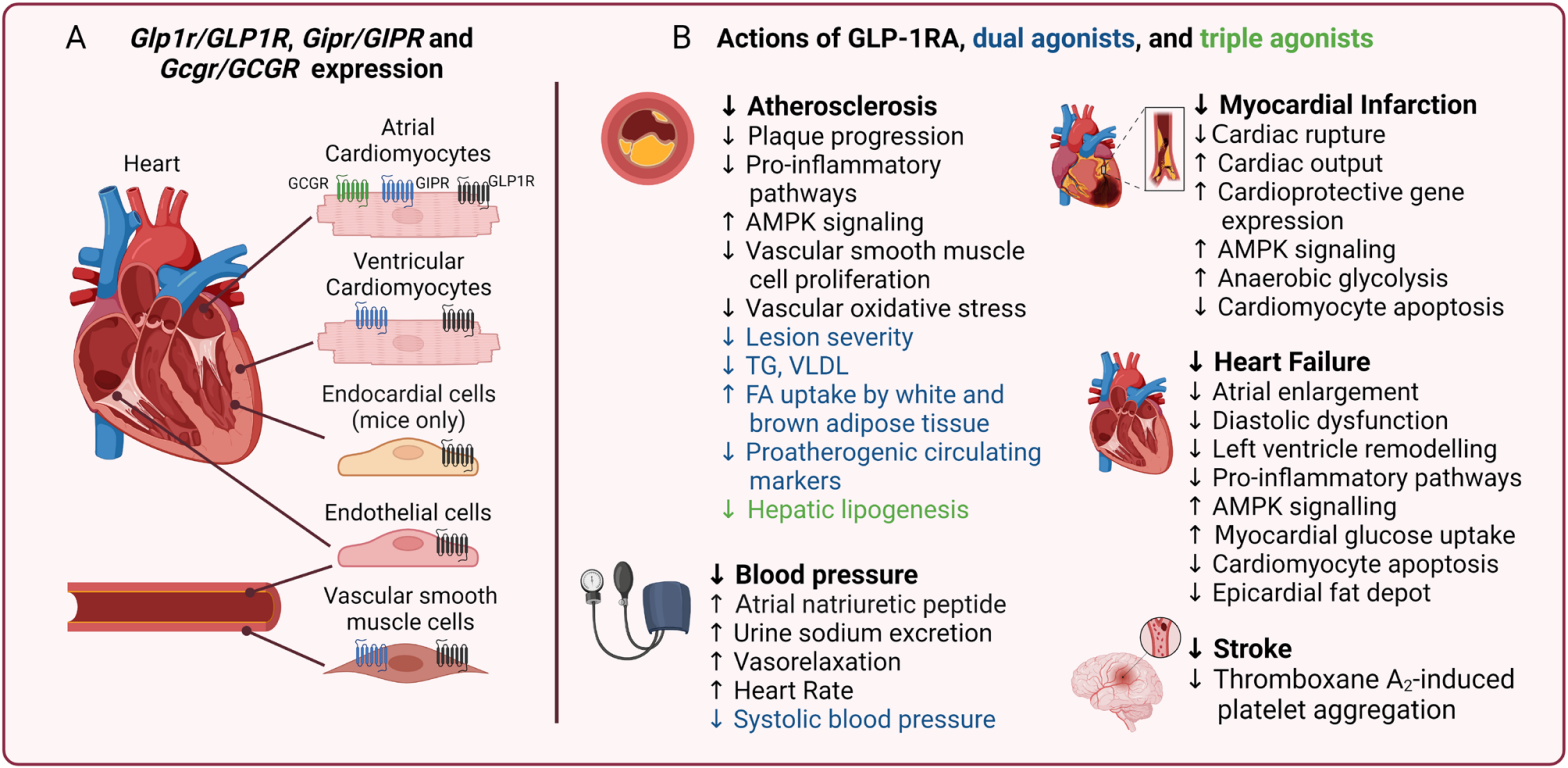
Cardiovascular benefits of GLP-1RA, dual, or triple agonists. (A) Distribution of *Glp1r or GLP1R* (in black), *Gipr* or *GIPR* (in blue), and *Gcgr* or *GCGR* (in green) mRNA in the heart of humans and mice. (B) Mechanisms of action by which GLP-1RA (black), dual (blue), or triple agonists (green) improve cardiovascular outcomes. GLP-1RA, glucagon-like peptide 1 receptor agonist; GIPR, glucose-dependent insulinotropic peptide receptor; GCGR, glucagon receptor; TG, triglycerides; VLDL, very low-density lipoproteins; AMPK, 5' adenosine monophosphate-activated protein kinase.

The GLP-1R is expressed in the heart and vasculature, suggesting that GLP-1R agonism may have direct actions on the cardiovascular system. However, species-specific differences in the cardiac distribution of murine *Glp1r* and human *GLP-1R* challenge the interpretation of mechanistic data (Baggio *et al.* 2018, McLean *et al.* 2021*a*,*b*). For instance, *Glp1r* mRNA transcripts were detected in atrial and ventricular adult mice cardiomyocytes (Kim *et al.* 2013), and single-cell RNA-seq analyses revealed that cardiac *Glp1r* is expressed in some endothelial cells and localized in the ventricular endocardium in mice (McLean *et al.* 2022). By contrast, RNA sequencing of normal and ischemic hearts from humans has identified *GLP1R* mRNA transcripts predominantly in a subset of atrial and ventricular cardiomyocytes, and they are relatively more abundant in human ventricles than in mice (McLean *et al.* 2022) (Fig. 1). However, definitive cellular localization of *GLP-1R* mRNA transcripts or immunoreactive GLP-1R protein within human cardiomyocytes remains elusive, as validated GLP-1R antisera lack sufficient sensitivity to detect the expression of endogenous cardiac GLP-1R by Western blotting. Therefore, although human ventricles express *GLP1R*, the identity of one or more ventricular cell type(s) that express the translated GLP1R protein requires further clarification with highly sensitive methods of detection (Baggio *et al.* 2018).

Unlike GLP-1R, *Gipr* and *GIPR* mRNA are widely expressed within the ventricular and atrial myocardium of both rodents and humans (Hiromura *et al.* 2016, Baggio *et al.* 2018), respectively, supporting a direct role of GIPR in cardiac function. However, mechanisms linking the action of GIP to cardiovascular outcomes remain unclear, especially in humans, as elevated GIP was found in individuals with atherosclerotic CVD (Kahles *et al.* 2018). Yet, human genetics associated GIP signaling with reduced body mass index (BMI), triglycerides, and lower rates of HF (Karhunen *et al.* 2021). The action of GIP on CVD in pre-clinical studies is also uncertain. GIPR agonism was shown to suppress macrophage-driven inflammation and foam cell formation in animal models of atherosclerosis, hypertensive cardiomyopathy, or MI (Nogi *et al.* 2012, Kahles *et al.* 2018, Terasaki *et al.* 2021). Some studies also reported that the loss of whole-body *Gipr* and selective loss of cardiomyocyte *Gipr* expression was reported to be cardioprotective in mice (Ussher *et al.* 2018), others reported that the loss of whole-body *Gipr* and selective loss of cardiomyocyte *Gipr* expression in MerCreMer transgenic mice expressing tamoxifen-inducible Cre was reported to be cardioprotective in mice (Pujadas *et al.* 2022). Whether these actions are conserved in humans, and depend on direct or indirect actions of GIP on the cardiovascular system, is currently unknown. Despite the debated role of GIP signaling in the cardiovascular system, the emergence of GLP-1R/GIPR dual agonists, such as tirzepatide, has resulted in greater improvements in T2DM and weight management than GLP-1RA alone (Lyons & Beaudry 2023). Whether this new class of medication shows superior cardioprotection is still under investigation. In the ongoing SURPASS-CVOT trial, the safety of tirzepatide versus dulaglutide is being assessed in patients living with T2DM with confirmed atherosclerotic CVD and is to be completed by 2025 (ClinicalTrials.gov ID NCT04255433).

The emergence of tri-agonists, combining GLP-1R/GIPR and glucagon (GCG) receptor (GCGR) agonists (GLP-1R/GIPR/GCGR agonist), has also shown promising results by leading to more significant weight loss, a greater percentage of individuals attaining weight loss, further lowering lipid levels, improving T2DM management, and enhancing overall substrate metabolism compared to the use of GLP-1R agonists alone (Lyons & Beaudry 2023). However, despite a significant examination of the physiology of GCGR signaling, the mechanisms underlying the impact of these new medications on cardiovascular health remain unclear. GCG is a peptide hormone secreted from pancreatic α-cells that stimulates hepatic glucose production, thereby maintaining adequate glycemia, but is also involved in hepatic lipid and amino acid metabolism and may increase resting energy expenditure (Habegger *et al.* 2010). Additionally, GCG is known for its inotropic effect, thereby enhancing the heart rate and contractility (Goldstein *et al.* 1971). This hormone signals by binding to and activating GCGR, which is a G protein-coupled receptor primarily expressed by hepatocytes in the liver. However, it is also expressed, to differing degrees, in the kidneys, adrenal glands, gastrointestinal tract, pancreas, adipose tissue, and heart (Svoboda *et al.* 1994, Neumann *et al.* 2023). *Gcgr* mRNA was found to be highly expressed in the murine right atrium, but weakly expressed in the left atrium or cardiac ventricle (Baggio *et al.* 2017). Interestingly, GCG was also shown to bind the GLP-1R, although it has about 140 times less affinity for GLP-1R than the specific agonists of these receptors (Chepurny *et al.* 2019). Interestingly, tri-agonists were shown to improve cardiovascular risk factors, such as decreasing systolic blood pressure and blood lipids in people living with obesity (Jastreboff *et al.* 2023) and T2DM (Rosenstock *et al.* 2023*a*,*b*). However, the interaction between the signaling circuits of these hormones is not yet fully elucidated and will require scrutiny to ascertain their cardiovascular safety.

This review will provide an overview of the data obtained from the CVOTs of GLP-1RAs and will discuss the latest cardiometabolic risk factor data from dual and tri-agonist therapies. We will then explore potential mechanisms that contribute to reducing major adverse cardiovascular events (MACE). Finally, we will discuss the current gaps and uncertainties that require further investigation.

### Cardiovascular outcome trials

Several multicenter, randomized, placebo-controlled trials have examined the impact of GLP-1R agonists on cardiovascular outcomes in patients with T2DM. The primary composite outcome in these trials included death from cardiovascular causes, non-fatal MI, and non-fatal stroke, collectively known as the three-point MACE. Eight trials have investigated the influence of GLP-1R agonists on MACE in T2DM patients to date, of which five showed favorable outcomes and three revealed neutral cardiovascular benefits. A more recent trial also demonstrated cardioprotective benefits in patients living with obesity but without T2DM.

The LEADER trial, which investigated the use of a 1.8 mg/day subcutaneous injection of liraglutide or placebo in 9340 patients with T2DM and high cardiovascular risk, was the first positive GLP-1RA CVOT trial published in 2016. The results were promising as the primary composite outcome (first occurrence of three-point MACE) occurred in 608 out of the 4668 patients (13.0%) treated with liraglutide as compared to 694 out of the 4672 patients (14.9%) in the placebo group. Also, the rates of death from cardiovascular causes in the liraglutide group were lower (219 patients (4.7%)) than in the placebo group (278 patients (6.0%)). Additionally, the rates of non-fatal MI, non-fatal stroke, and hospitalization for HF tended to be lower in the liraglutide group compared to the placebo group (Marso *et al.* 2016*b*). This was the first trial to demonstrate that among patients with T2DM at high risk for CVD, liraglutide decreased the rates of cardiovascular events and death from any cause compared to the placebo group. The SUSTAIN-6 trial then investigated the use of 0.5 mg or 1.0 mg once-weekly subcutaneous injection of semaglutide in 3297 patients with T2DM for 2 years. The study found that patients who received semaglutide were less likely to experience major cardiovascular events. Specifically, the primary composite outcome occurred in 108 out of 1648 patients (6.6%) in the semaglutide group, whereas 146 out of 1649 patients (8.9%) in the placebo group experienced the same outcome. Semaglutide also reduced the incidence of non-fatal stroke and tended to decrease non-fatal MI but did not reduce the rate of death from cardiovascular causes (Marso *et al.* 2016*a*). The Harmony Outcomes Trial, conducted in 2018, investigated the use of 30–50 mg albiglutide once-weekly subcutaneous injection in 9463 patients with T2DM for a median of 1.5 years. Albiglutide significantly reduced the incidence of three-point MACE in patients with T2DM and CVD. The incidence of three-point MACE was 7% (338 events) in the albiglutide group compared to 9% (428 events) in the placebo group. The reduction primarily reflected a significant decrease in non-fatal MI rates (Hernandez *et al.* 2018). In 2019, the REWIND trial found that in the 9901 patients with T2DM with and without previous CVD randomly assigned to a once-weekly 1.5 mg dulaglutide injection or placebo (median follow-up 5.4 years), three-point MACE occurred in 594 patients receiving dulaglutide (12.0%) and in 663 patients (13.4%) in the placebo group, which included a significant decrease in the rate of non-fatal stroke (Gerstein *et al.* 2019).

More recently, the AMPLITUDE-O trial sought to examine the effect of efpeglenatide, an exendin-based GLP-1RA that is structurally dissimilar to other GLP-1RAs examined in cardiovascular outcome trials. In this study, 4076 patients living with T2DM and either a history of CVD (90%) or kidney disease and at least one other cardiovascular risk factor were randomized to receive once-weekly 4 mg or 6 mg efpeglenatide injection or placebo (median follow-up 1.81 years). Efpeglenatide significantly decreased the rate of three-point MACE (3.9%) compared to placebo (5.3%). This was accompanied by a decreased incidence of an expanded MACE composite endpoint (Gerstein *et al.* 2021).

While results from LEADER, SUSTAIN-6, Harmony Outcomes, REWIND, and AMPLITUDE-O have shown a decrease in three-point MACE in T2DM patients, differences in the benefits have been observed as clinical practice has evolved over this time. The AMPLITUDE-O trial, which included more high-risk patients and had a higher prevalence of kidney disease than the previous GLP-1RA CVOT trials, observed an overall lower incidence of at least one MACE composite event. SUSTAIN-6 reported a reduction in non-fatal stroke, while the Harmony Outcomes trials observed a reduction in non-fatal MI, and the LEADER trial observed fewer deaths from cardiovascular causes in the liraglutide group than in the placebo group. These differences may arise due to variations in the duration of treatment, the extent of GLP-1R engagement, trial design, inclusion and exclusion criteria, and differences in the populations studied. For instance, in the REWIND trial, only 31.5% of patients had CVD, while in the other trials, approximately 70% of enrolled patients had CVD, and 60–70% were men. Importantly, the use of DPP4 inhibitors was considered an exclusion criterion in the LEADER and SUSTAIN-6 trials, but not in Harmony Outcomes and REWIND. In the AMPLITUDE-O trial, 15% of patients included were also receiving sodium-glucose cotransporter 2 (SGLT2) inhibitors, yet efpeglenatide decreased the rate of MACE irrespective of whether patients were also receiving this treatment.

While five studies have found a positive impact of GLP1R agonists on cardiovascular outcome trials in patients living with T2DM, three have found neutral effects. For instance, the ELIXA trial investigated the impact of lixisenatide, a short-acting GLP-1RA, on 6068 patients with T2DM who had an acute coronary event within 3 months before screening. The study concluded that lixisenatide did not impact the composite of death from cardiovascular causes, non-fatal MI, non-fatal stroke, or hospitalization for unstable angina (with a median follow-up of 25 months) (Pfeffer *et al.* 2015). Similarly, the EXSCEL trial showed that the incidence of three-point MACE with 2 mg once-weekly exenatide was not significantly different from placebo in 14,752 patients living with T2DM (median follow-up of 3.2 years) (Holman *et al.* 2017). In contrast, the FREEDOM trial reported that the constant infusion of exenatide (20 ug daily for 3 months followed by 60 µg daily) through an osmotic minipump was non-inferior to the placebo in 4156 T2DM patients (with a median follow-up of 16 months) (Ruff *et al.* 2022). Finally, the PIONEER 6 trial demonstrated that the use of a 14 mg daily dose of oral semaglutide in a population with T2DM and high cardiovascular risk was non-inferior to that of placebo. The median time in the trial was 15.9 months, and 3.8% of the semaglutide group experienced the primary outcome, compared to 4.8% in the placebo, despite significant differences in weight loss and glycated hemoglobin reduction (Husain *et al.* 2019). The hazard ratios were similar in the PIONEER 6 and SUSTAIN-6 trials, suggesting that the cardiovascular effects of semaglutide are independent of the route of administration.

Understanding the lack of improved cardiovascular outcomes with GLP-1R agonists in ELIXA, EXSCEL, PIONEER 6, and FREEDOM CVOTs is complex, potentially originating from differences in trial design, variable sample sizes, discontinuation rates, follow-up durations, and challenges in achieving sustained pharmacokinetics during drug development. In the EXSCEL trial, the discontinuation rate was higher than 40% in both groups. Exenatide and lixisenatide, displaying less robust GLP-1R target engagement, exhibit lower effectiveness for glucose control and weight loss compared to dulaglutide, liraglutide, and semaglutide (Trujillo *et al.* 2021, Viljoen & Bain 2023). Nonetheless, in the EXSCEL trial, once-weekly exenatide demonstrated a significant 12% reduction in all-cause mortality and nearly significant decreases in three-point MACE.

More recently, the SELECT trial investigated whether semaglutide could reduce cardiovascular risk in individuals with overweight or obesity and preexisting CVD, but not T2DM. In this trial, 17,604 patients were assigned to receive a once-weekly injection of 2.4 mg of semaglutide or placebo (mean follow-up of 39.8 months). The primary cardiovascular end-point event occurred in 569 of the 8803 patients (6.5%) in the semaglutide group and 701 of the 8801 patients (8.0%) in the placebo group (Lincoff *et al.* 2023). This is the first trial to demonstrate the cardiovascular benefits of semaglutide in patients with preexisting CVD and overweight or obesity but without T2DM.

### Heart failure reported in CVOTs

Another critical endpoint assessed during the GLP-1RA CVOTs is hospitalization for HF (Marso *et al.* 2016*a*,*b*, Hernandez *et al.* 2018, Gerstein *et al.* 2019, 2021). A meta-analysis of the ELIXA, LEADER, SUSTAIN-6, EXSCEL, Harmony Outcomes, REWIND, PIONEER 6, and AMPLITUDE-O trials involving over 60,000 patients with T2DM demonstrated an 11% decrease in HF hospitalization (Sattar *et al.* 2021). Approximately 60% of patients with HF with preserved ejection fraction (HFpEF) also live with obesity (Morgen *et al.* 2023), which is accompanied by greater symptom severity, reduced exercise capacity, more adverse hemodynamics, and a greater risk of hospitalization for HF than those living with HFpEF without obesity (Borlaug *et al.* 2023*a*). Recently, the STEP-HFpEF trial sought to examine the results of 2.4 mg weekly treatment with semaglutide for 52 weeks on various outcomes related to HFpEF and obesity. Semaglutide treatment reduced body weight significantly and improved symptoms, physical limitations, and exercise function in patients across all classes of obesity. A reduction in C-reactive protein (CRP) levels was seen across all classes of obesity. The amelioration of symptoms as assessed by the Kansas City Cardiomyopathy Questionnaire-Clinical Summary Score, and improvements in exercise function seemed to be directly related to the magnitude of weight loss, such that greater improvement was observed with greater body weight loss (Borlaug *et al.* 2023*b*). Furthermore, in a trial examining the effect of 2.4 mg semaglutide once weekly in people with obesity and HFpEF and T2DM, it was demonstrated that semaglutide led to a larger reduction in HF-related symptoms, physical limitations, and weight loss, and greater improvements in exercise than placebo at 1 year (Kosiborod *et al.* 2024) (Table 1).

**Table 1:** Interventions, comparators, and key outcomes from GLP-1RA clinical trials for CVD outcomes.

Trial	Population	Intervention	Comparison	Outcome
LEADER (60)	Age ≥50 years with T2DM and established CVD or age >60 years with cardiovascular risk factors (*n* = 9340)	Once-daily 1.8 mg liraglutide subcutaneous injection for median 3.8 years	Volume-matched placebo subcutaneous injection once-daily	↓ MACE, ↓ death from cardiovascular cause, ↓ death from any cause
SUSTAIN-6 (61)	Age ≥50 years with T2DM and established CVD or age >60 years with cardiovascular risk factors (*n* = 3297)	Once-weekly 0.5 or 1.0 mg semaglutide subcutaneous injection for 104 weeks	Volume-matched placebo subcutaneous injection once-weekly	↓ MACE, ↓ non-fatal stroke
Harmony Outcomes (62)	Age ≥40 years with T2DM and established CVD (*n* = 9463)	Once-weekly 30–50 mg albiglutide subcutaneous injection for median 1.5 years	Volume-matched placebo subcutaneous injection once-weekly	↓ MACE, ↓ fatal or non-fatal MI
REWIND (63)	Age ≥50 years with T2DM and a previous cardiovascular event or cardiovascular risk factor (*n* = 9901)	Once-weekly 1.5 mg dulaglutide subcutaneous injection for median 5.4 years	Volume-matched placebo subcutaneous injection once-weekly	↓ MACE
AMPLITUDE-O (64)	Adults with T2DM and history of CVD or >50 (if male) or >55 (if female) years with kidney disease and at least one cardiovascular risk factor (*n* = 4076)	Once-weekly 4–6 mg efpeglenatide subcutaneous injection for median 1.81 years	Volume-matched placebo subcutaneous injection once-weekly	↓ MACE
SELECT (71)	Age ≥45 years with BMI >27 and established cardiovascular disease (*n* = 17,604)	Once-weekly 2.4 mg semaglutide subcutaneous injection for mean follow-up 39.8 months	Volume-matched placebo subcutaneous injection once-weekly	↓ MACE
ELIXA (65)	Age ≥30 years with T2DM and an acute coronary event within the previous 180 days (*n* = 6068)	Once-daily 10–20 µg lixisenatide subcutaneous injection for a median 25 months	Volume-matched placebo subcutaneous injection once-daily	↔ MACE
EXSCEL (66)	Adults with T2DM with or without previous cardiovascular events (*n* = 14,752)	Once-weekly 2 mg extended-release exenatide subcutaneous injection for median 3.2 years	Volume-matched placebo subcutaneous injection once-daily	↔ MACE
FREEDOM (67)	Adults with T2DM with or at risk for atherosclerotic cardiovascular disease (*n* = 4156)	Continuous subcutaneous injection exenatide for median 16 months	Volume-matched subcutaneous injection of placebo	↔ MACE
PIONEER-6 (68)	Age ≥50 years with T2DM and established CVD or chronic kidney disease or age >60 years with cardiovascular risk factors (*n* = 3183)	Once-daily oral 14 mg semaglutide for a median 15.9 months	Matched daily oral placebo	↔ MACE

BMI, body mass index; MACE, major adverse cardiovascular event; T2DM, type 2 diabetes.

### Dual-agonist clinical trials

Finan *et al.* (2013) provided an early indication in rodents, primates, and humans that combining GLP-1R and GIPR agonists could be more effective than administering GLP-1RA alone. Their study found that simultaneous administration of equimolar amounts of these agonists for 2 weeks reduced food intake, body weight, and fat mass in mice with diet-induced obesity to a greater extent than either agent alone. While many studies have shown that dual agonists (GLP-1R/GIPR agonists) and tri-agonists (GLP-1R/GIPR/GCGR agonists) improve glycemia and body weight in amounts unprecedented by the single agents (Nauck & D'Alessio 2022), only a few have investigated their role in cardiovascular outcomes. Although there is not a lot of data to support the use of dual and triple agonists for hard cardiovascular outcomes, the available trials suggest that the use of these therapies improves cardiometabolic risk factors, such as reducing blood pressure, triglycerides, and low-density lipoprotein cholesterol (LDL-C). Inflammatory markers like CRP and circulating adhesion molecules like ICAM-1 were also reduced by tirzepatide treatment compared to dulaglutide and placebo in a phase 2 trial with patients living with T2DM (Wilson *et al.* 2022). Yet, more studies are needed to confirm this observation and to elucidate the underlying mechanisms.

The SURMOUNT program is a series of global, randomized design, phase 3 clinical trials investigating the effect of the GLP-1R/GIPR dual-agonist tirzepatide in obese and overweight patients with a mean age of 44.9–54.2 years, mostly female (50.7–69.7%), and with a BMI of 36.1–38.9 kg/m^2^. Although these trials focus on obesity management, the population recruited was either at risk of weight-related complications or at high risk for CVD. Participants from the SURMOUNT-1 trials living with overweight or obesity without diabetes randomized to receive 15 mg tirzepatide weekly lost a mean of 20.9% of their body weight compared to baseline (Jastreboff *et al.* 2022), while those in the SURMOUNT-2 living with overweight or obesity with T2DM lost on average 14.7% (Garvey *et al.* 2023). In the two trials, improvements in all prespecified cardiometabolic measures, including waist circumference, systolic and diastolic blood pressure, fasting insulin, lipid, and aspartate aminotransferase levels, were observed with tirzepatide (Jastreboff *et al.* 2022, Garvey *et al.* 2023). In the SURMOUNT-3 trial, patients living with obesity or overweight without diabetes or pre-diabetes who had lost >5% of body weight (roughly 7.6 kg) following a 12-week intensive lifestyle intervention were randomized to placebo or maximally tolerated tirzepatide dose (10 or 15 mg). After 72 weeks, patients in the tirzepatide group lost 18.4% of their body weight, while those in the placebo group experienced a 2.5% increase. Improvement in lipid parameters was also demonstrated in the tirzepatide group, which may be due to the significant weight loss (Wadden *et al.* 2023). Finally, in the SURMOUNT-4 trial, after 36 weeks of the maximum tolerated dose of tirzepatide (10 or 15 mg), an adult living with overweight or obesity without diabetes experienced a mean weight reduction of 20.9%. From randomization at week 36, those who switched to placebo experienced a 14% weight regain and those continuing tirzepatide lost an additional 5.5% weight following the 52-week double-blind period. Relative to placebo, tirzepatide was also associated with significant improvements in BMI, HbA1c, fasting glucose, fasting insulin, lipid levels, and systolic and diastolic pressure, which emphasizes the need to continue pharmacotherapy to prevent weight regain and ensure the maintenance of cardiometabolic benefits (Aronne *et al.* 2024) (Table 2).

**Table 2:** Interventions, comparators, and key outcomes from tirzepatide clinical trials for CVD risk factors.

Trial	Population	Intervention	Comparison	Outcome
SURMOUNT-1 (80)	Adults with BMI ≥30 or ≥27 with at least one weight-related complication and one or more unsuccessful dietary efforts to lose weight (*n* = 2539)	Once-weekly 5, 10, or 15 mg tirzepatide subcutaneous injection for 72 weeks	Volume-matched placebo subcutaneous injection once-weekly	↓ Weight (−15.0%, −19.5%, and −20.9% for 5, 10, and 15 mg, respectively), ↓ SBP, ↓ DBP, ↓ TG, ↓ non-HDL-C, ↓ fasting insulin
SURMOUNT-2 (81)	Adults with BMI ≥27 with T2DM (*n* = 1514)	Once-weekly 10 or 15 mg tirzepatide subcutaneous injection for 72 weeks	Volume-matched placebo subcutaneous injection once-weekly	↓ Weight (−12.8% and −14.7% for 10 and 15 mg, respectively), ↓ HbA1c, ↓ TG, ↓ non-HDL-C, ↑ HDL, ↓ LDL-C, ↓ VLDL-C, ↓ total cholesterol, ↓ FFA
SURMOUNT-3 (82)	Adults with BMI ≥30 or ≥27 with at least one weight-related complication (excluding T2DM) who achieved ≥5.0% weight reduction after a 12-week intensive lifestyle intervention (*n* = 579)	Once-weekly 10 or 15 mg tirzepatide subcutaneous injection for 72 weeks	Volume-matched placebo subcutaneous injection once-weekly	↓ Weight (−18.4% with maximum tolerated dose of tirzepatide), ↓ SBP, ↓ DBP, ↓ TG, ↓ non-HDL-C, ↑ HDL, ↓ LDL-C, ↓ VLDL-C, ↓ total cholesterol, ↓ FFA, ↓ fasting insulin
SURMOUNT-4 (83)	Adults with BMI ≥30 or ≥27 and at least one weight-related complication (excluding T2DM) receiving once-weekly 10 or 15 mg tirzepatide subcutaneous injection for 36 weeks (*n* = 783)	Continue with once-weekly 10 or 15 mg tirzepatide subcutaneous injection for another 36 weeks	Volume-matched placebo subcutaneous injection once-weekly	↓ Weight (− 20.9% with tirzepatide from weeks 0 to 36; −5.5% with tirzepatide and +14.0% with placebo from weeks 36 to 88; −25.3% with tirzepatide from weeks 0 to 88)
SURPASS-1 (84)	Adults (18–75 years) with T2DM and BMI of 25–50 kg/m^2^ (*n* = 281)	Once-weekly 0.5 mg, 4 mg escalation, 4 mg, 8 mg slow escalation, 8 mg fast escalation, 12 mg escalation injection for 24 weeks	1.5 mg dulaglutide or volume-matched placebo injections once-weekly	↓ Weight, ↓ TG, ↓ non-HDL cholesterol, ↓ SBP, ↓ DBP
SURPASS-2 (85)	Adults with T2DM inadequately controlled with metformin at a dose of at least 1500 mg per day (*n* = 1879)	Once-weekly 5, 10, or 15 mg tirzepatide subcutaneous injection for 72 weeks	1 mg semaglutide subcutaneous injection once-weekly	↓ HbA1c, ↓ weight, ↓ TG, ↓ VLDL-C, ↓ SBP, ↓ DBP, ↑ HDL-C
SURPASS-3 (86)	Adults with T2DM, BMI > 25, stable weight, insulin-naive, and treated with metformin alone or in combination with an SGLT2 inhibitor for at least 3 months before screening (*n* = 1444)	Once-weekly 5, 10, or 15 mg tirzepatide subcutaneous injection for 52 weeks	Once-daily subcutaneous injection of titrated insulin degludec	↓ HbA1c, ↓ weight, ↓ SBP, ↓ DBP, ↓ TG, ↓ VLDL-C, ↑ HDL-C,
SURPASS-4 (87)	Adults with T2DM treated with any combination of metformin, sulfonylurea, or SGLT2 inhibitor, BMI ≥ 25, and established cardiovascular disease or a high risk of cardiovascular events (*n* = 2002)	Once-weekly 5, 10, or 15 mg tirzepatide subcutaneous injection for 52 weeks	Glargine (100 U/mL) titrated to reach fasting blood glucose of less than 100 mg/dL	↓ HbA1c, ↔ MACE compared to glargine
SURPASS-5 (88)	Adults with T2DM and inadequate glycemic control while treated with once-daily insulin glargine with or without metformin (*n* = 475)	Once-weekly 5, 10, or 15 mg tirzepatide subcutaneous injection for 40 weeks	Volume-matched placebo subcutaneous injection once-weekly	↓ HbA1c, ↓ weight, ↓ total cholesterol, ↓ LDL-C, ↓ VLDL-C, ↓ TG
SURPASS-6 (89)	Adults with T2DM inadequately controlled with basal insulin with or without any combination of up to 2 of the following oral glucose-lowering medications: metformin, sulfonylurea, DPP-4 inhibitors (*n* = 1428)	Once-weekly 5, 10, or 15 mg tirzepatide subcutaneous injection for 40 weeks	Thrice daily prandial insulin lispro	↓ HbA1c, ↓ weight, ↓ SBP, ↓ DBP, ↓ TG, ↓ total cholesterol, ↓ LDL-C, ↓ VLDL-C, ↓ non-HDL-C, ↑ HDL

BMI, body mass index; DBP, diastolic blood pressure; FFA, free fatty acid; HbA1c, glycated hemoglobin; HDL-C, high-density lipoprotein cholesterol; LDL-C, low-density lipoprotein cholesterol; MACE, major adverse cardiovascular event; SBP, systolic blood pressure; SGLT2, sodium-glucose transport protein 2; T2DM, type 2 diabetes; TG, triglyceride; VLDL-C, very-low-density lipoprotein cholesterol.

Likewise, five clinical trials in T2DM patients (SURPASS 1–5) have shown that tirzepatide at 5–15 mg per week reduces both HbA1c (1.24–2.58%) and body weight (5.4–11.7 kg) by amounts unprecedented for a single agent (Del Prato *et al.* 2021, Frias *et al.* 2021*c*, Ludvik *et al.* 2021, Rosenstock *et al.* 2021, Dahl *et al.* 2022). Cardiovascular events have been adjudicated across the whole study program, and incidences of non-fatal MI, non-fatal stroke, cardiovascular death, and hospital admission for angina tended to be reduced over up to 2 years, albeit with low numbers of events (Nauck & D'Alessio 2022). Systolic blood pressure was reduced in all SURPASS trials in a dose-dependent manner, by approximately 5–6 mm Hg (Del Prato *et al.* 2021, Frias *et al.* 2021*c*, Ludvik *et al.* 2021, Rosenstock *et al.* 2021, Dahl *et al.* 2022), more substantially than with semaglutide, which yielded a 3.6 mm Hg decrease on average (Frias *et al.* 2021*c*). Furthermore, in the SURPASS-1 and -2 studies, a significant reduction in TG by 18.5–24.8%, in low-density lipoprotein cholesterol (LDL-C) by 5.2–12.4%, and in very low-density lipoprotein (VLDL)-cholesterol by 17.5–23.7%, as well as a significant increase of high-density lipoprotein cholesterol (HDL-C) by 3.2–7.9% were confirmed (Frias *et al.* 2021*c*, Rosenstock *et al.* 2021). Improvement in triglycerides and cholesterol using tirzepatide significantly surpassed those observed with 1.0 mg/week of semaglutide (Frias *et al.* 2021*c*). In the recently published SURPASS-6 trial, 1428 patients with T2DM using basal insulin were randomized to either prandial insulin Lispro, 5 mg, 10 mg, or 15 mg of once-weekly tirzepatide for 52 weeks. Lispro was given prandially, three times a day to achieve a pre-lunch, pre-dinner, and bedtime blood glucose target of 100–125 mg/dL (5.6–6.9 mmol/L). Despite similar baseline characteristics, the tirzepatide groups showed significant reductions in HbA1c% with a pooled average of 68% of participants meeting the established criteria of 7.0% compared to 36% in the insulin Lispro group. The average lowering of HbA1c was 2.11% in the tirzepatide group and 1.13% in the insulin Lispro group. The tirzepatide group experienced a significant weight loss benefit as well, with a pooled reduction of 9 kg, compared to the insulin group which experienced a 3.2 kg weight gain over 52 weeks. The dose escalation of tirzepatide produced greater weight loss and greater HbA1c reduction compared to insulin Lispro. Given the results, the addition of tirzepatide to basal insulin in patients with inadequately controlled T2DM resulted in a substantial lowering of HbA1c (Rosenstock *et al.* 2023*a*,*b*). This, however, cannot be uncoupled from the weight loss produced by tirzepatide based on the current study design.

Other ongoing trials are also investigating the impact of tirzepatide on cardiovascular safety and efficacy. The SURMOUNT-MMO trial (ClinicalTrials.gov ID NCT05556512), estimated to be completed in October 2027, is currently studying the role of tirzepatide in people with a BMI ≥27 kg/m^2^ without diabetes, with a high risk for CVD. In this event-driven cardiovascular efficacy trial, lasting up to 5 years, participants will be monitored for the first occurrence of non-fatal MI or stroke, coronary revascularization, HF events, and mortality. In the SUMMIT trial (ClinicalTrials.gov ID NCT04847557), estimated to have been completed in November 2023 but not published yet, people with obesity and HFpEF received tirzepatide for 52 weeks. The primary outcome is a hierarchical composite endpoint comprising mortality, HF events, exercise capacity, and HF symptoms. In the ongoing SURPASS-CVOT trial (ClinicalTrials.gov ID NCT04255433), the safety of tirzepatide versus GLP-1RA dulaglutide is assessed in patients living with T2DM with confirmed atherosclerotic CVD, including participants treated with insulin and SGLT2 inhibitors, where the composite primary outcome will be the time to first occurrence of MACE.

### Triple agonist clinical trials

Recent studies using tri-agonists targeting GLP-1R, GIPR, and GCGR have demonstrated impressive outcomes (Coskun *et al.* 2022). In a phase 2 trial conducted by Jastreboff *et al.*, 338 adults with a BMI >30 or higher or a BMI >27 plus at least one weight-related condition (hypertension, dyslipidemia, or CVD) were randomly assigned to receive subcutaneous injections of the tri-agonist retatrutide (1 mg, 4 mg (initial dose, 2 mg), 4 mg (initial dose, 4 mg), 8 mg (initial dose, 2 mg), 8 mg (initial dose, 4 mg), or 12 mg (initial dose, 2 mg)) or placebo once weekly for 48 weeks. The primary endpoint was the percentage change in body weight from baseline to 24 weeks. Secondary endpoints included the percentage change in body weight from baseline to 48 weeks and a weight reduction of 5% or more, 10% or more, or 15% or more. Retatrutide reduced body weight by 24.2% from baseline at 48 weeks (12 mg high dose). In a subgroup of participants with BMI of ≥35 kg/m^2^, high doses of 8 and 12 mg of retatrutide effected a 26.5% weight loss over 48 weeks compared to those categorized with BMI <35 kg/m^2^, who achieved 22.1%. Moreover, 100% of participants treated with 8 or 12 mg of retatrutide achieved a targeted body weight loss >5%, as compared to 27% in the placebo group. Interestingly, female participants lost more weight than male participants over the 48 weeks, achieving 28.5% and 21.9% with 12 mg, respectively. Significant improvements were observed in blood pressure, HbA1c, and lipid profiles, though HDL-C showed no significant change. Improvements in blood pressure within the 48-week treatment period resulted in the discontinuation of at least one antihypertensive medication in 41% of the participants in the combined 8 mg group and 30% of the participants in the 12 mg group. The weight loss observed in this trial significantly exceeds the weight loss observed in previous trials, likely due to the triple receptor action of retatrutide (Jastreboff *et al.* 2023) (Table 3).

**Table 3:** Interventions, comparators and key outcomes from retatrutide clinical trials for CVD risk factors.

Trial	Population	Intervention	Comparison	Outcome
Jastreboff *et al.*2023 (58)	Adults with BMI ≥ 30 with at least one weight-related condition (*n* = 338)	Once-weekly 1 mg, 4 mg (initial dose 2 mg), 4 mg (initial dose 4 mg), 8 mg (initial dose 2 mg), 8 mg (initial dose 4 mg), 12 mg (initial dose 2 mg subcutaneous injections for 48 weeks	Volume-matched placebo subcutaneous injection once-weekly	↓ Weight, ↓ HbA1c, ↓ SBP, ↓ DBP, ↓ fasting glucose, ↓ insulin, ↓ TG, ↓ total cholesterol, ↓ LDL-C, ↓ VLDL-C
Rosenstock *et al.* 2023*a*(59)	Adults (18–75 years) with T2DM and BMI 25–50 kg/m^2^ (n = 281)	Once-weekly 0.5 mg, 4 mg escalation, 4 mg, 8 mg slow escalation, 8 mg fast escalation, 12 mg escalation injection for 36 weeks	1.5 mg dulaglutide or volume-matched placebo injections once-weekly	↓ HbA1c, ↓ weight, ↓ triglycerides, ↓ non-HDL-C, ↓ SBP, ↓ DBP

BMI, body mass index; DBP, diastolic blood pressure; HbA1c, glycated hemoglobin; HDL-C, high-density lipoprotein cholesterol; LDL-C, low-density lipoprotein cholesterol; SBP, systolic blood pressure; T2DM, type 2 diabetes; TG, triglyceride; VLDL-C, very-low-density lipoprotein cholesterol.

In a phase 2 trial, Rosenstock *et al.* recruited 281 participants with T2DM living with overweight or obesity who were randomized to receive once-weekly injections of placebo, 1.5 mg of GLP-1RA dulaglutide, or tri-agonist retatrutide maintenance doses of 0.5 mg, 4 mg (starting dose 2 mg), 4 mg (no escalation), 8 mg (starting dose 2 mg), 8 mg (starting dose 4 mg), or 12 mg (starting dose 2 mg). The authors observed a dose-dependent body weight reduction of up to 16.9% with retatrutide (12 mg escalating group) at 36 weeks, which did not appear to have plateaued at 36 weeks, versus 3.0% with the placebo and 2% with 1.5 mg dulaglutide. This magnitude of body weight reduction had not been reported to date in any other phase 2 or 3 trials testing weekly GLP-1RA or GLP-1R/GIPR dual agonists in patients living with T2DM, potentially due to the additional agonism of the glucagon receptor (Grunberger *et al.* 2012, Nauck *et al.* 2016, Frias *et al.* 2018, Davies *et al.* 2021, Frias *et al.* 2021*a*,*b*,*c*). An HbA1c reduction of 2.02% was observed for the 12 mg escalation group after 36 weeks, compared to a 0.01% reduction for the placebo and a 1.41% reduction for the 1.5 mg dulaglutide group. Concurrently with reductions in glycemia and body weight, retatrutide dose-dependently reduced systolic and diastolic blood pressure and improved lipid profiles, notably reducing non-HDL-C concentrations while decreasing triglyceride by up to 35% (Rosenstock *et al.* 2023*a*,*b*). Subsequent longitudinal studies involving a larger and more diverse global study population will enhance our understanding of the enduring corrective effects of retatrutide, particularly in relation to cardiovascular health outcomes.

As of now, it is not possible to uncouple the potential benefit of the co-agonists from the additional weight loss. Nevertheless, mechanistic data support the putative cardiovascular benefits from GLP-1R agonism in spite of less understood effects of other receptors.

### Mechanistic data

Delineating the mechanisms underlying the cardiovascular benefits of GLP-1RA, dual agonists (GLP-1R/GIPR agonists), and tri-agonists (GLP-1R/GIPR/GCGR agonists) is complex, given that their receptors are expressed in various cells across the organism (Hammoud & Drucker 2023). While the mechanisms linking the action of GIP and GCG to cardiovascular outcomes remain unclear, many studies have shown that GLP-1RA could improve cardiovascular outcomes by acting on the central nervous system to decrease appetite and inflammation (Wong *et al.* 2024), ultimately leading to decreased adiposity and body weight (Drucker 2022), decreasing hepatic steatosis and circulating lipid levels (Targher *et al.* 2023), or by preserving renal function (Cherney *et al.* 2021). This section will focus on the mechanisms that may be at play in the cardiovascular system, with particular attention given to preclinical studies examining the potential benefits of GLP-1RA, as well as emerging evidence supporting the role of dual and triple agonist therapies.

### Atherosclerosis and GLP-1RA

The clinical trials investigating the impact of GLP-1RA on cardiovascular health have shown that the incidence of MACE does not differ between the GLP-1R agonist and placebo groups until 12–18 months after initiation of therapy, which aligns with the potential anti-atherogenic effect of GLP-1RA (Sattar *et al.* 2021). Preclinical models commonly used for evaluating CVD include atherosclerosis-prone mice with genetic elimination of apolipoprotein E (*ApoE*^−/^^−^ mice) and of the low-density lipoprotein receptor *(Ldlr^−/^*^−^ mice*).* These models are favored due to their heightened susceptibility to atherosclerotic plaque lesions, especially when subjected to a high-fat, high-cholesterol diet (Getz & Reardon 2016, Fuchs & Whelton 2020). Administration of semaglutide and liraglutide in both mouse models fed a Western diet for 12–17 weeks led to significantly attenuated plaque progression, which was independent of weight and cholesterol-lowering. Semaglutide decreased plasma levels of markers of systemic inflammation in an acute inflammation model (lipopolysaccharide treatment), and transcriptomic analysis of aortic atherosclerotic tissue revealed that multiple inflammatory pathways were downregulated (Rakipovski *et al.* 2018). In 6–9 weeks old *ApoE*^−/−^ mice fed a Western diet, daily administration of liraglutide for up to 12 weeks reduced plaque burden, decreased the expression of vascular cell adhesion molecule (VCAM-1) three-fold compared to placebo, as measured by contrast-enhanced ultrasound molecular imaging, and reduced plasma TNF-α, IL-1β, MCP-1, and osteopontin, a glycophosphoprotein that partly acts as a cytokine. No changes in LDL-C and HbA1c were observed, suggesting that GLP-1RAs affect atherosclerosis through an anti-inflammatory mechanism (Punjabi *et al.* 2023).

The reduction in vessel lesion formation observed with GLP-1RA might also be linked to cellular signaling within the vessel walls. *ApoE*^−/^^−^ mice treated with liraglutide 400 µg/day for 4 weeks exhibited increased AMP-activated protein kinase (AMPK) phosphorylation in the aortic wall, as measured by immunohistochemistry. In the same study, it was discovered that *ex vivo* liraglutide not only increased AMPK signaling but also inhibited angiotensin II-induced proliferation of vascular smooth muscle cells by arresting the cell cycle in the G0/G1 phase in a GLP-1R-dependent manner, thus delaying the progression of atherosclerosis independently of its glucose-lowering effect (Jojima *et al.* 2017). To further elucidate which GLP-1R-expressing cells are involved in the abovementioned cardio- and vasoprotective benefits, liraglutide was administered to wild-type (C57BL/6J), global *Glp1r* knockout (*Glp1r*^−/^^−^), endothelial cell- (*Cdh5^Cre^*) and myeloid cell (*LysM^Cre^*)-specific knockout of *Glp1r* and arterial hypertension was induced by angiotensin II. Liraglutide normalized vascular fibrosis, endothelial dysfunction, oxidative stress, and vascular inflammation. More precisely, liraglutide prevented leukocyte rolling on the endothelium and infiltration of myeloid Ly6G^−^Ly6C^+^ and Ly6G^+^Ly6C^+^ cells into the vascular wall, which resulted in decreased vascular oxidative stress, reduced S-glutathionylation, a marker of endothelial nitric oxide synthase (eNOS) uncoupling, and increased nitric oxide (NO) bioavailability. Importantly, the positive cardiovascular effects of liraglutide were sustained in mice with myeloid cell *Glp1r* deficiency but were eliminated in *Glp1r^−/^*^−^ mice and endothelial cell-specific *Glp1r* knockout mice (Helmstadter *et al.* 2020), indicating that GLP-1R activation in endothelial cells by liraglutide achieves these cardiovascular benefits. Hence, these investigations illustrate that GLP-1R agonism effectively slows down the advancement of atherosclerosis through a glycemia-independent and endothelial GLP-1R-dependent pathway. This positions GLP-1RAs as promising candidates for averting or postponing the development of vessel plaque accumulation and inflammation in individuals at risk of CVD.

### Blood pressure and GLP-1RA

Many preclinical studies have demonstrated that GLP-1 and GLP-1RAs reduce BP in experimental animal models of hypertension (Kim *et al.* 2013, Simonds *et al.* 2019, Helmstadter *et al.* 2020). For example, treatment with exendin-4 for 12 weeks in salt-sensitive obese *db/db* mice prevented the development of hypertension, despite increased intra-renal angiotensin II levels (Hirata *et al.* 2009). Liraglutide was shown to increase the secretion of postprandial atrial natriuretic peptide (ANP) from cardiomyocytes and increased urine sodium excretion in wild-type mice, but did not induce ANP secretion, vasorelaxation, or lower blood pressure in *Glp1r^−/^*^−^ or *Nppa^−/^*^−^ mice (Kim *et al.* 2013). Likewise, in the abovementioned study by Helmstadter, Frenis *et al.*, liraglutide administered to mice with hypertension induced by angiotensin II also normalized blood pressure, which resulted in reduced cardiac hypertrophy and a reduced heart/body weight ratio, both parameters indicative of hypertensive heart disease. Mechanistically, GLP-1R activation by liraglutide attenuated vessel wall infiltration with inflammatory monocytes and neutrophils, which in turn lowered oxidative stress and prevented uncoupling of eNOS. With NO bioavailability maintained, endothelium-mediated vasorelaxation was preserved and vascular remodeling and fibrosis were prevented (Helmstadter *et al.* 2020). The blood pressure lowering effect observed in this study was consistent with previous data (Kim *et al.* 2013), but with a much greater magnitude than what is typically observed in human studies, which report a 2–4 mm Hg reduction (Nauck *et al.* 2017). Exendin-4 prevented angiotensin II-induced hypertension in non-diabetic mice and inhibited angII-induced phosphorylation of ERK1/2 in cultured renal cells. Considered together, these results suggest that exendin-4 has anti-hypertensive effects through the attenuation of angiotensin II-induced high-salt sensitivity (Hirata *et al.* 2009). Although both preclinical and clinical studies have shown that liraglutide increases the secretion of ANP from atrial cardiomyocytes (Kim *et al.* 2013, Li *et al.* 2014), this effect has been observed inconsistently across clinical trials (Lovshin *et al.* 2015). It is also worth noting that a degradation-resistant GLP-1RA often fails to improve endothelial or vascular function in human studies to the same levels as observed in preclinical studies (Kelly *et al.* 2012, Nandy *et al.* 2014, Ussher *et al.* 2014), which suggests that a more comprehensive evaluation of GLP-1R expression and function in endothelial and vascular smooth muscle cells is needed.

GLP-1RAs are also known to increase heart rate. Short-acting GLP-1RAs like exenatide and lixisenatide are associated with transient (1–12 h) increases, causing a maximum heart rate increase of 8–10 beats per minute (bpm) 2–4 h after injection, which returned to baseline after approximately 10–12 h, with a mean 24 h heart rate increase of approximately 1–2 bpm for both GLP-1RAs in healthy adults (https://www.glucagon.com/pdfs/ByettaCanadaPM_11Jan2011_pswd.pdf; Linnebjerg *et al.* 2011). On the other hand, long-acting GLP-1RAs are associated with more pronounced increases; liraglutide and albiglutide increased the heart rate by 6–10 bpm, while dulaglutide and long-acting release exenatide increased heart rate by a mean 3–4 bpm in 24 h in healthy adults (Darpo *et al.* 2013, 2014, Ferdinand *et al.* 2014; https://www.accessdata.fda.gov/drugsatfda_docs/nda/2019/206321Orig1s004.pdf). In a population of adults with overweight or obesity and T2DM, liraglutide was associated with a mean increase in heart rate of 3 bpm (Marso *et al.* 2016*b*). The underlying mechanism for increased heart rate remains to be elucidated but is hypothesized to be related to a direct effect at the sinus node and/or stimulation of the sympathetic nervous system, with this effect related to the duration of action of the respective GLP-1RAs (Lorenz *et al.* 2017). However, regardless of the magnitude, GLP-1RA-increased heart rate does not present an increased cardiovascular risk for subjects with T2DM (Lorenz *et al.* 2017).

### Myocardium and GLP-1RA

Preclinical studies demonstrated that GLP-1RAs protect against myocardial ischemia-reperfusion injury in rats (Bao *et al.* 2011, Aravindhan *et al.* 2015), mice (Noyan-Ashraf *et al.* 2009), and pigs (Timmers *et al.* 2009). Administration of liraglutide to normal and diabetic mice before inducing MI reduced cardiac rupture and infarct size, improved cardiac output, and modulated the expression of cardioprotective genes in the mouse heart, including Akt, GSK-3beta, PPARbeta-delta, NRF-2, and HO-1. The cardioprotective and survival advantages of liraglutide were superior to those of metformin in both normal and diabetic mice and were independent of weight loss (Noyan-Ashraf *et al.* 2009). Likewise, liraglutide activated several cardioprotective pathways which prevented insulin resistance and inflammation, decreased vascular adhesions, and improved ventricular function by activating AMPK, independent of changes in body weight in mice fed a high-fat diet (Noyan-Ashraf *et al.* 2013). Interestingly, administration of liraglutide in mice with selective disruption of the cardiomyocyte GLP-1R (*Glp1r^CM−/^^−^*) generated from MerCreMer transgenic mice expressing tamoxifen-inducible Cre driven by the α-myosin heavy chain promoter, still produced robust cardioprotection and increased survival, suggesting that cardiomyocyte GLP-1R activation is not required for adaptive responses to ischemic and cardiomyopathic injury mediated by liraglutide (Ussher *et al.* 2014, Scott *et al.* 2016). By contrast, the deletion of *Glp1r* in Tie2+ endothelial cells (*Glp1rTie2^−^/^−^*) attenuated the cardioprotective actions of liraglutide to reduce infarct size, increase ejection fraction, and improve survival after experimental MI (McLean *et al.* 2022), indicating that GLP-1R signaling in endothelial cells contributes to the cardioprotective effect of liraglutide in mice with ischemic cardiac injury. GLP-1R activation using liraglutide was protective against diabetic cardiomyopathy in a mouse model of T2DM, likely due to an improved response to insulin at the cardiac level and increased glycolytic enzyme activity. Interestingly, despite evidence for the GLP-1R in cardiac tissue, the effects of liraglutide were observed following systemic administration of liraglutide and not through direct treatment of the heart. This provides evidence that cardiovascular benefits from GLP-1R agonism may also come from metabolic changes in cardiac tissue through systemic signaling (Almutairi *et al.* 2021).

The GLP-1R agonist-mediated protection against ischemic heart disease was shown to require caveolin, which is a membrane scaffolding protein regulating protective signaling and myocyte ultrastructure in the setting of ischemic stress (Schilling *et al.* 2015). Administration of exendin-4 to mice subjected to simulated ischemia/reperfusion decreased infarct size and cardiac troponin, but the cardioprotective effects were abolished in caveolin-3 knockout mice (Tsutsumi *et al.* 2014). This could potentially be linked to the ability of GLP-1RA to enhance microvasculature blood circulation. Indeed, GLP-1(7-36)amide infusion improved musculature blood flow and increased muscle glucose uptake via an NO-dependent mechanism in the vascular endothelium (Chai *et al.* 2012, Dong *et al.* 2013). In line with this, albiglutide was shown to enhance myocardial glucose uptake and a shift toward a more energetically favorable substrate metabolism by increasing both glucose and lactate oxidation in rats subjected to 30-min myocardial ischemia followed by 24-h reperfusion. The switch to anaerobic glycolysis in the ischemic area provided a compensatory substrate switch to overcome the energetic deficit in this region with reduced tissue oxygenation. In contrast, a switch to more energetically favorable carbohydrate oxidation in more highly oxygenated remote regions supported maintaining cardiac contractility in a complementary manner (Bao *et al.* 2011, Aravindhan *et al.* 2015). Another identified mechanism supporting the protective role of GLP-1 against myocardial ischemia/reperfusion injury is the reduction of cardiomyocyte apoptosis, as evidenced by the phosphorylation and inactivation of the pro-apoptotic peptide BAD in GLP-1-treated rat hearts (Bose *et al.* 2005).

### Stroke and GLP-1RA

Putative mechanisms linking GLP-1R activation to reduced rates of stroke are still emerging but are likely multifactorial with indirect effects on platelet aggregation. Two weeks of liraglutide administration (0.6 mg/d for 1 week, 1.2 mg/d for 1 week, then 1.8 mg/d subcutaneously) in patients with obesity significantly reduced Thromboxane A_2_-induced platelet aggregation from baseline (Cahill *et al.* 2022). These findings were associated with platelet binding to LUXendin645, a fluorescent GLP-1R antagonist (Ast *et al.* 2020), *ex vivo*. Platelet-specific GLP-1R expression and function were confirmed using the selective GLP-1R antagonist exendin(9-39), supporting a GLP-1R-dependent action of GLP-1RA on platelets (Cahill *et al.* 2022). A pilot study in patients with T2DM receiving liraglutide (1.8 mg/day for 6 months) also demonstrated a transient and significant attenuation in the maximum slope of platelet aggregation in response to collagen, arachidonic acid, and adenosine diphosphate (Loganathan *et al.* 2022). However, a more detailed analysis to determine whether human platelets express a functional canonical GLP-1R that can transduce a sustained decrease in platelet aggregation is still warranted. Meta-analyses have demonstrated that despite an overall benefit, some heterogeneity exists between GLP-1RAs and their effect on stroke. Although the studies were not powered for stroke outcomes alone, a particularly large benefit was seen with exenatide and dulaglutide use in the EXSCEL and REWIND trials, despite exenatide results not being significant. This contrasts with albiglutide, which had very little effect on stroke outcomes (Bellastella *et al.* 2020, Sattar *et al.* 2021).

One possible explanation is that the effect of GLP-1RAs on stroke outcomes is produced through a combination of central and peripheral mechanisms, potentially associated with weight loss and blood pressure, as albiglutide did not produce substantial weight loss or systolic blood pressure differences.

A retrospective analysis in an American population with T2DM found that GLP-1RA usage was associated with a significantly lower risk of stroke than DPP4i. Both groups had similar baseline characteristics, although neither glycated hemoglobin nor fasting blood glucose measurements were available, thereby making it difficult to gauge whether both groups had equally advanced T2DM (Evans *et al.* 2024).

### Heart failure and GLP-1RA

HF can occur after MI, which damages the heart muscle and reduces its ability to contract. Many preclinical models have recapitulated the protective effect of GLP-1RA observed in humans (Borlaug *et al.* 2023*b*) in experimental HF models to elucidate the underlying mechanisms (Nikolaidis *et al.* 2004, Poornima *et al.* 2008, Noyan-Ashraf *et al.* 2009, Bhashyam *et al.* 2010, Noyan-Ashraf *et al.* 2013, Sassoon *et al.* 2017). For instance, in aged high-fat diet-fed female mice with experimental HFpEF induced by angiotensin II infusion, liraglutide treatment (1 mg/kg once daily for 12 weeks) was shown to alleviate cardiac hypertrophy, atrial enlargement, and diastolic dysfunction, as evidenced by increased global longitudinal strain (Withaar *et al.* 2021). Pretreatment with liraglutide for one week before left anterior descending (LAD) coronary artery occlusion significantly improved the survival of C57BL/6J mice (Noyan-Ashraf *et al.* 2009).

Several mechanisms have been proposed to support the GLP-1R-dependent improvement in HF. In a mouse model of obesity-induced cardiomyopathy, liraglutide treatment decreased left ventricle (LV) remodeling and improved LV stroke volume, which was associated with decreased myocardial expression of the proinflammatory marker TNFα and nuclear localization of nuclear factor-κB (Noyan-Ashraf *et al.* 2013). Another mechanism could be linked to increased AMPK activity, which can not only improve the energy supply in the failing heart by promoting ATP production but can also regulate several important physiological processes to restore heart function (Li *et al.* 2019). In the study by Noyan-Ashraf *et al.*, liraglutide improved measures of cardiac performance through an AMPK-dependent mechanism (Noyan-Ashraf *et al.* 2013). Administration of GLP-1(7-36) amide was also shown to stimulate myocardial glucose uptake in dogs with dilated cardiomyopathy, which improved both left ventricular and systemic hemodynamics (Nikolaidis *et al.* 2004, Bhashyam *et al.* 2010). Likewise, spontaneous hypertensive HF-prone rats treated with exogenous GLP-1 exhibited prolonged survival and were associated with preserved LV function and LV mass index, increased myocardial glucose uptake, and decreased myocyte apoptosis (Poornima *et al.* 2008). GLP-1RA, such as liraglutide, semaglutide, or dulaglutide was also shown to decrease the epicardial fat depot in preclinical models and humans, which is known to cause mechanical constriction of the diastolic filling and is a source of pro-inflammatory mediators (Salvatore *et al.* 2022). Liraglutide was also shown to decrease epicardial fat depot thickness (29% at 3 months and 36% at 6 months) in patients with T2DM randomized to liraglutide with metformin compared to patients only receiving metformin (Iacobellis *et al.* 2017). Finally, a reduction in cardiomyocyte apoptosis was also noted in response to GLP-1RA treatment, as evidenced by the decreased expression level of cleaved caspase 3 in the hearts of spontaneous hypertensive HF-prone rats (Poornima *et al.* 2008) and mice with LAD coronary artery occlusion (Noyan-Ashraf *et al.* 2009).

### Mechanistic data on GIPR agonists and dual agonists

Despite the inclusion of GIPR agonism in dual and triple agonists, the mechanistic effects of GIPR agonism on cardiovascular outcomes are not entirely understood (Heimburger *et al.* 2020). Some evidence points toward both acute and chronic treatment with GIP increasing markers of inflammation and upregulating transcripts and proteins associated with inflammatory pathways in adipocytes and mouse models of obesity (Timper *et al.* 2013, Chen *et al.* 2015) Seemingly contradictory data have indicated that systemic use of a long-acting form of GIP, [d-Ala2]GIP, reduces inflammatory cytokine expression in adipose tissue and decreases the number of circulating monocytes and neutrophils in a mouse model of obesity (Varol *et al.* 2014). Kim *et al.* (2012) demonstrated that genetic overexpression of GIP in mice resulted in a favorable lowering of pro-inflammatory gene expression in adipocytes, as well as resistance to diet-induced obesity and improvements in glucose homeostasis. While the effects of GIPR agonism may be confounded by other anti-inflammatory variables such as weight loss and improvements in glucose homeostasis, GIP unquestionably has an important, albeit enigmatic, role in immune signaling. Compared to a saline intraperitoneal injection, GIP release was higher following exposure to LPS in mice, and in an IL-6 and IL-1-dependent fashion, with GIP antagonism blunting the inflammatory response. In mice, the potentiating effects on GLP-1, glucagon, and insulin secretion also seemed to be dependent on IL-6 (Kahles *et al.* 2016, Timper *et al.* 2016). Despite the nebulous relationship between GIP and inflammation, this evidence may suggest that increased inflammation is necessary for GIP signaling, but that greater systemic changes precipitated by GIP action may produce a net reduction in inflammatory markers.

One preclinical study compared the effects of single GIPR or GLP-1R agonism and combined GLP-1R/GIPR agonism in female APOE*3-Leiden.CETP mice fed a Western-type diet, a well-established mouse model for human-like lipoprotein metabolism and atherosclerotic development, for 10 weeks. While single GLP-1R agonism (GLP-140) or GIPR agonism (GIPFA-085) only resulted in non-significant improvements in atherosclerosis, they observed that dual agonism reduced lesion severity in APOE*3-Leiden.CETP mice and circulating triglyceride levels. Lower triglyceride levels were associated with decreases in the production of VLDL particles as well as increased triglyceride-derived fatty acid uptake by both brown and white adipose tissue. Combined GIPR/GLP-R agonism additionally lowered CD36 levels in circulating monocytes compared to the vehicle treatment (van Eenige *et al.* 2023). CD36 is considered a biomarker for atherosclerosis lesion progression as positive associations have been found between *Cd36* mRNA expression in the aortic wall and in peripheral blood mononuclear cells, where it stimulates inflammatory responses and foam cell formation (Yazgan *et al.* 2018, Zhao *et al.* 2018). While this study was only performed in relatively lean female mice, which are mostly protected against diet-induced obesity, it is in line with a study performed in patients receiving tirzepatide, dulaglutide, or placebo, which showed that tirzepatide treatment dose-dependently decreased levels of ApoC-III and ApoB and the number of large triglyceride-rich lipoprotein particles and small LDL particles, suggesting a net improvement in atherogenic lipoprotein profile (Wilson *et al.* 2020). Nevertheless, more studies in various models of cardiometabolic disease are needed to confirm the cardioprotective mechanisms, especially considering that there is limited evidence available to elucidate the mechanistic details regarding the cardiovascular biology or long-term safety of selective GIPR agonism in humans (Hammoud & Drucker 2023).

### Mechanistic data on GLP-1R/GIPR/GCGR tri-agonists

The combination of GLP-1RA, GIPRA, and GCGRA in preclinical studies has demonstrated superior weight loss relative to liraglutide in mice with diet-induced obesity, attributed to decreased food intake and increased energy expenditure (Baggio & Drucker 2021). Examinations of a tri-agonist in *Gcgr*^−/^^−^ mice demonstrated the retention of glucoregulatory effects but a significant reduction in the weight loss outcomes. This emphasized the crucial role of GCGR activation in the metabolic actions of the tri-agonist. Notably, when an acylated GCGR agonist was administered alone, it led to approximately 20% weight loss in mice on a high-fat diet, and this weight loss was further enhanced when the GCGR agonist was combined with a GLP-1R/GIPR co-agonist. Consequently, these findings underscore the significance of GCGR activation in achieving weight loss in preclinical studies (Finan *et al.* 2015). GLP-1R/GCGR agonism with cotadutide was also shown to indirectly improve experimental components of steatohepatitis through direct actions to reduce hepatic lipogenesis and increase mitochondrial oxidative capacity, in addition to weight loss (Boland *et al.* 2020)

While there have been improvements in cardiometabolic factors in response to GCGR activation such as body weight, steatohepatitis, and glucose homeostasis, there are concerns due to the potential impact of GCGR agonism on the heart. GCG has the capacity to influence factors like the force of contraction, beating rate, and alterations in the cardiac conduction system axis. However, evidence regarding hemodynamic effects is limited and inconsistent. Additionally, the understanding of the mechanisms of GCGR agonists is complicated by its potential activation of the GLP-1R (Neumann *et al.* 2023). In a mouse model of MI, an antibody against glucagon improved cardiac function and ameliorated the progression of HF by blunting cardiac hypertrophy, fibrotic remodeling, and attenuated contractile dysfunction at 4 weeks after MI (Gao *et al.* 2019). In addition, glucagon may contribute, at least partly, to the development of diabetic cardiomyopathy, as antagonism of the GCGR reduced its incidence in a mouse model of diabetes over several months (Sharma *et al.* 2018). Furthermore, increased glucagon levels may be harmful due to the possible chronic tachycardia (Chang-Chretien *et al.* 2004, Zhang *et al.* 2014), which is a risk factor for cardiovascular morbidity (Palatini *et al.* 1997). Safety concerns were also raised in a phase 1 trial investigating the GLP-1R/GCGR agonist NNC9204-117, where a dose-dependent increase in heart rate was observed, in addition to increased markers of inflammation, hepatic disturbances, impaired glucose tolerance, and reduced blood levels of some amino acids despite clinically relevant weight loss (Friedrichsen *et al.* 2022). Finally, concerns regarding the use of tri-agonism in patients have centered around the potential of GCGR agonism to elevate heart rate, in addition to its effects on increasing energy expenditure (Petersen *et al.* 2018). However, recent findings with retatrutide indicate that while heart rate peaks after 24 weeks of treatment in individuals with obesity, it subsequently decreases beyond 36 weeks (Jastreboff *et al.* 2023). These results are comparable to those observed in individuals receiving GLP-1R agonists and the co-agonists tirzepatide and retatrutide for the treatment of T2DM (Lorenz *et al.* 2017, Frias *et al.* 2021*c*, Rosenstock *et al.* 2023*a*,*b*). Future longitudinal studies involving a larger and more diverse global study population are needed to further clarify the long-term corrective effects of triple agonists such as retatrutide, particularly on cardiovascular health outcomes.

### Future directions

GLP-1R agonists, as well as dual and tri-agonists, are generally considered safe and effective, though they often cause side effects like nausea, vomiting, constipation, and diarrhea (Drucker & Holst 2023). Efforts are underway to improve tolerance, with new long-acting GIPR agents enhancing the gastrointestinal tolerability of GLP-1RA therapy (Knop 2023). Despite these side effects, GLP-1RAs and multi-agonists offer a therapeutic alternative that has the potential to reach a broad population at high cardiometabolic risk. In line with this, oral GLP-1RAs such as semaglutide and orforglipron are available, providing a non-invasive and effective approach to managing body weight, blood sugar, and other cardiovascular risk factors (Wharton *et al.* 2023, Furusawa *et al.* 2024). Furthermore, an oral version of a GLP-1R/GIPR agonist is undergoing phase 2 trials (ClinicalTrials.gov ID NCT06068946). While these oral treatments are less invasive, one major drawback of GLP-1RA-based therapies is their high cost. Additionally, long-term pharmacotherapy might be necessary, as stopping medication often leads to a loss of GLP-1RA benefits, even with continued lifestyle changes (Rubino *et al.* 2021, Wilding *et al.* 2022). Yet, although GLP-1RA is more expensive than other glucose-lowering agents, recent cost-effectiveness analyses suggest that in US patients with T2D and a CVD-related hospitalization, the added medical cost of treatment with GLP-1RAs is offset by lower inpatient and outpatient care costs, resulting in budget neutrality against standard of care (Evans *et al.* 2022).

## Conclusion

In summary, the collective evidence from various studies, including those exploring dual agonists (GLP-1R/GIPR) and tri-agonists (GLP-1R/GIPR/GCGR), demonstrates unprecedented improvements in diabetes and body weight compared to GLP-1RA treatment alone. Despite these positive effects, their impact on cardiovascular outcomes has not been largely explored. Although the current data is insufficient to strongly advocate for the cardiovascular benefits of dual and triple agonists, ongoing trials suggest potential improvements in cardiometabolic risk factors, such as reduced blood pressure, triglycerides, and LDL-C levels. However, further comprehensive studies are crucial to validate these preliminary findings and unveil the underlying mechanisms. In particular, the consequences of weight regain following a ~20% reduction in body weight on the cardiovascular system must be explored. Moreover, while there is substantial evidence supporting the cardioprotective role of GLP-1RA, the data is less conclusive for GIP and GCG, necessitating additional research to clarify their contributions to cardiovascular health.

## Declaration of interest

The authors declare that there is no conflict of interest that could be perceived as prejudicing the impartiality of the research reported.

## Funding

This work did not receive any specific grant from any funding agency in the public, commercial, or not-for-profit sector.
